# Supplementary figures and images for: Gut microbiome signatures in iNPH: Insights from a shotgun metagenomics study
Source: PLoS One. 2025 Sep 15;20(9):e0330251. doi: 10.1371/journal.pone.0330251 (PMC12435679; doi:10.1371/journal.pone.0330251)

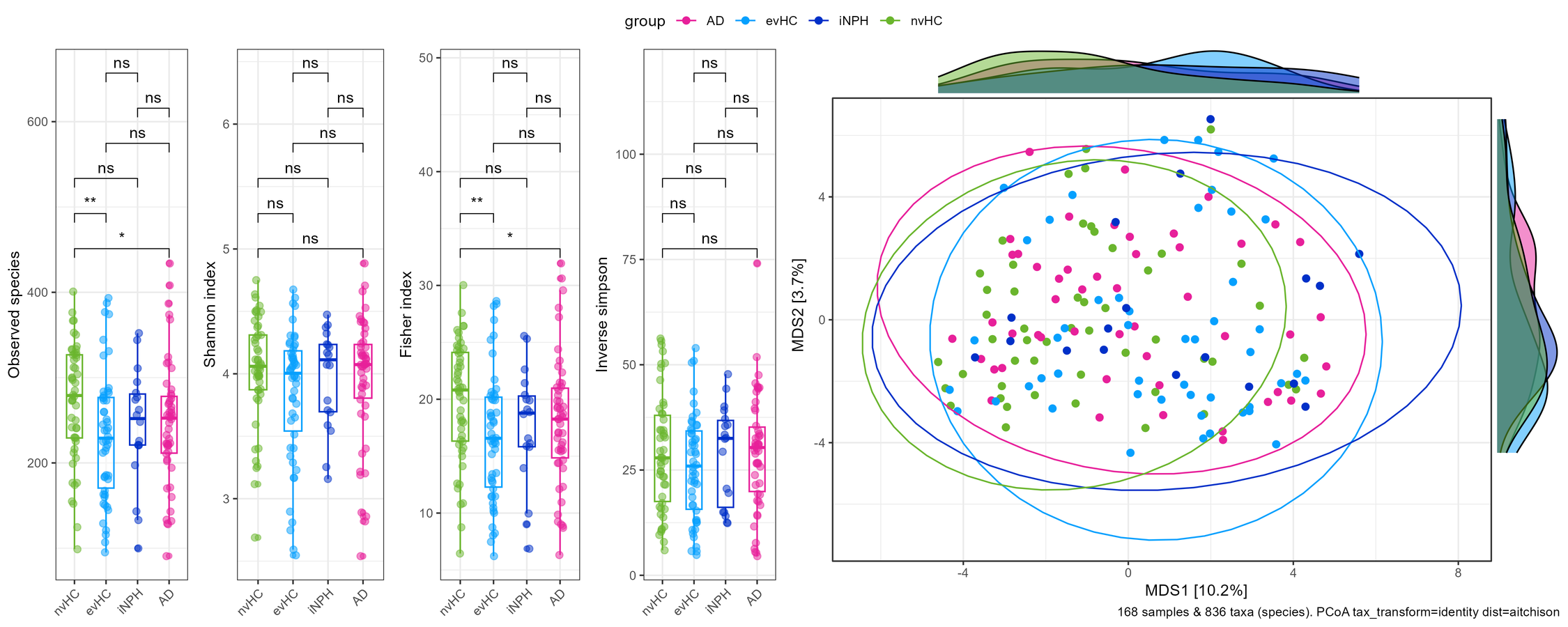

Supplement: S1 Fig — Boxplots showing the alpha diversity comparisons between study groups (A). Principal Coordinates Analysis (PCoA) showing the distribution of bacterial communities across study groups, dataset was filtered for 5% minimum prevalence (B). (TIF) [file pone.0330251.s001.tif]
