# Supplementary material for: Gut microbiome signatures in iNPH: Insights from a shotgun metagenomics study
Source: PLoS One. 2025 Sep 15;20(9):e0330251. doi: 10.1371/journal.pone.0330251 (PMC12435679; doi:10.1371/journal.pone.0330251)
Supplement: S1 File — (DOCX) [file pone.0330251.s002.docx]

**Supplementary tables.**

**Table S1.** Comparison of characteristics of eligible evHC participants: accepted vs. not accepted.

| **Accepted** | no | yes | p-value |
| --- | --- | --- | --- |
| **Total** | 80 | 56 |  |
| **Age** | 73.5 ± 7.5 | 70.1 ± 7.6 | 0.011 |
| **Female** | 53.8 | 57.1 | 0.829 |
| **Years of education** | 13.3 ± 4.2 | 14.9 ± 3.9 | 0.022 |
| **Mini Mental State Exam** | 27.5 ± 1.4 | 28.2 ± 1.4 | 0.007 |
| **Average MTA visual rating** | 1.5 ± 0.9 | 1.5 ± 0.7 | 0.844 |

**Table S2. PERMANOVA analysis of microbiome composition.** Variables were evaluated for their effect on the study population microbiome. R^2^ represents the proportion of variance explained by the factors included in the analysis. P-values were adjusted with Benjamini-Hochberg method. ** adjusted p < 0.05 , n.s. not significant*

| ***Variable*** | | ***β diversity distance*** | | ***R2*** | ***p-value*** | ***adjusted p*** |
| --- | --- | --- | --- | --- | --- | --- |
| *Number of reads* | | Bray-curtis | | 0.006 | 0.407 | n.s. |
|  |  | Aitchison | | 0.0068 | 0.155 | n.s. |
| *Gender* | | Bray-curtis | | 0.0089 | 0.016 | n.s. |
|  |  | Aitchison | | 0.0111 | 0.002 | * |
| *Age* | | Bray-curtis | | 0.0074 | 0.101 | n.s. |
|  |  | Aitchison | | 0.0055 | 0.611 | n.s. |
| *Body mass index* | | Bray-curtis | | 0.0105 | 0.004 | n.s. |
|  |  | Aitchison | | 0.0074 | 0.061 | n.s. |
| *Study group* | | Bray-curtis | | 0.0211 | 0.069 | n.s. |
|  |  | Aitchison | | 0.0251 | 0.001 | * |
|  |  |  |  |  |  |  |

**Table S3. Combined taxonomic results from Maaslin2 and ANCOM-BC differential abundance analysis.** Group comparisons with nvHC as the reference. Covariates included gender and BMI.

| feature | q-value evHC Maaslin2 | q-value iNPH Maaslin2 | q-value evHC ancombc | q-value iNPH ancombc | Direction |
| --- | --- | --- | --- | --- | --- |
| *Blautia_SGB101324* | 0.6982 | 0.1863 | 1.0000 | 0.5166 | Decreased in evHC and/or iNPH compared to nvHC |
| *Clostridiaceae_bacterium_Marseille_Q4145* | 0.4205 | 0.1778 | 1.0000 | 1.0000 |  |
| *Clostridium_sp_AF12_28* | 0.0240 | 0.2638 | 0.0143 | 1.0000 |  |
| *Clostridium_sp_AF20_17LB* | 0.5445 | 0.1761 | 1.0000 | 1.0000 |  |
| *Collinsella_aerofaciens* | 0.5386 | 0.1849 | 1.0000 | 1.0000 |  |
| *Faecalibacterium_sp_CLA_AA_H233* | 0.0508 | 0.3347 | 0.4291 | 1.0000 |  |
| *Fusicatenibacter_saccharivorans* | 0.6982 | 0.1761 | 1.0000 | 1.0000 |  |
| *GGB33469_SGB15236* | 0.1686 | 0.0501 | 1.0000 | 0.8157 |  |
| *GGB4585_SGB6340* | 0.2352 | 0.1135 | 1.0000 | 0.0034 |  |
| *GGB9614_SGB15049* | 0.1270 | 0.3207 | 0.8124 | 1.0000 |  |
| *GGB9642_SGB15119* | 0.5243 | 0.1761 | 1.0000 | 1.0000 |  |
| *GGB9758_SGB15368* | 0.0039 | 0.4825 | 0.0027 | 1.0000 |  |
| *Oscillibacter_sp_ER4* | 0.1426 | 0.3207 | 1.0000 | 1.0000 |  |
| *Ruminococcus_lactaris* | 0.4467 | 0.1778 | 1.0000 | 1.0000 |  |
| *Anaerotruncus_colihominis* | 0.1761 | 0.2104 | 0.7628 | 1.0000 | Increased in evHC and/or iNPH compared to nvHC |
| *Anaerotruncus_massiliensis* | 0.7164 | 0.0073 | 1.0000 | 0.0186 |  |
| *Blautia_wexlerae* | 0.6635 | 0.4417 | 1.0000 | 0.1137 |  |
| *Butyricimonas_faecalis* | 0.1778 | 0.2497 | 0.2625 | 1.0000 |  |
| *Christensenella_hongkongensis* | 0.1863 | 0.0459 | 0.2123 | 0.1564 |  |
| *Clostridium_scindens* | 0.1808 | 0.4698 | 1.0000 | 1.0000 |  |
| *Cuneatibacter_caecimuris* | 0.1761 | 0.0117 | 0.1047 | 0.3324 |  |
| *Cuneatibacter_sp_NSJ_177* | 0.1761 | 0.0007 | 0.1573 | 0.0063 |  |
| *Dielma_fastidiosa* | 0.3400 | 0.1026 | 1.0000 | 0.3887 |  |
| *Diplocloster_agilis* | 0.2104 | 0.0025 | 1.0000 | 0.0005 |  |
| *Eisenbergiella_tayi* | 0.3194 | 0.0501 | 1.0000 | 0.0489 |  |
| *Enterocloster_bolteae* | 0.1135 | 0.0117 | 0.3077 | 0.0003 |  |
| *Enterocloster_clostridioformis* | 0.1824 | 0.5821 | 1.0000 | 1.0000 |  |
| *Eubacteriaceae_bacterium_Marseille_Q4139* | 0.1024 | 0.3347 | 0.0844 | 1.0000 |  |
| *Evtepia_gabavorous* | 0.4552 | 0.0501 | 1.0000 | 0.0006 |  |
| *Flavonifractor_plautii* | 0.1761 | 0.2522 | 1.0000 | 0.7460 |  |
| *GGB2982_SGB3964* | 0.6769 | 0.1761 | 1.0000 | 0.2856 |  |
| *GGB4583_SGB6334* | 0.0451 | 0.8998 | 0.0594 | 1.0000 |  |
| *GGB58233_SGB79883* | 0.1761 | 0.2703 | 0.5959 | 1.0000 |  |
| *GGB9522_SGB98319* | 0.3621 | 0.1135 | 1.0000 | 1.0000 |  |
| *GGB9719_SGB53514* | 0.8960 | 0.1686 | 1.0000 | 1.0000 |  |
| *Guopingia_tenuis* | 0.5866 | 0.1778 | 1.0000 | 0.5074 |  |
| *Hungatella_hathewayi* | 0.1976 | 0.0970 | 1.0000 | 0.2805 |  |
| *Intestinimonas_butyriciproducens* | 0.4628 | 0.1761 | 1.0000 | 0.2752 |  |
| *Lachnotalea_sp_AF33_28* | 0.2898 | 0.1849 | 1.0000 | 0.8384 |  |
| *Lactococcus_lactis* | 0.7483 | 0.0392 | 1.0000 | 0.4062 |  |
| *Massiliimalia_timonensis* | 0.5336 | 0.1849 | 1.0000 | 1.0000 |  |
| *Ruminococcus_gnavus* | 0.2898 | 0.1761 | 1.0000 | 0.0994 |  |
| *Ruthenibacterium_lactatiformans* | 0.4907 | 0.2322 | 1.0000 | 0.0179 |  |

**Table S4. Abundance and prevalence of the differentially abundant taxa**

| *Species* | nvHC abundance (%) | nvHC prevalence (%) | evHC abundance (%) | evHC prevalence (%) | iNPH abundance (%) | iNPH prevalence (%) | AD abundance (%) | AD prevalence (%) |
| --- | --- | --- | --- | --- | --- | --- | --- | --- |
| *Blautia SGB101324* | 0.045 ± 0.124 | 30 | 0.035 ± 0.117 | 14 | 0 ± 0 | 0 | 0.008 ± 0.032 | 8 |
| *Clostridiaceae bacterium Marseille Q4145* | 0.026 ± 0.046 | 56 | 0.013 ± 0.036 | 36 | 0.01 ± 0.026 | 22.2 | 0.012 ± 0.018 | 52 |
| *Clostridium sp AF12 28* | 0.021 ± 0.048 | 52 | 0.002 ± 0.008 | 12 | 0.01 ± 0.027 | 22.2 | 0.023 ± 0.08 | 30 |
| *Clostridium sp AF20 17LB* | 0.061 ± 0.086 | 64 | 0.044 ± 0.099 | 44 | 0.007 ± 0.015 | 27.8 | 0.042 ± 0.075 | 54 |
| *Collinsella aerofaciens* | 0.167 ± 0.279 | 86 | 0.228 ± 0.864 | 68 | 0.217 ± 0.421 | 50 | 0.286 ± 0.461 | 66 |
| *Faecalibacterium sp CLA AA H233* | 0.32 ± 0.532 | 74 | 0.082 ± 0.202 | 38 | 0.179 ± 0.381 | 44.4 | 0.19 ± 0.496 | 56 |
| *Fusicatenibacter saccharivorans* | 0.438 ± 0.43 | 96 | 0.326 ± 0.359 | 90 | 0.243 ± 0.233 | 72.2 | 0.335 ± 0.355 | 90 |
| *GGB33469 SGB15236* | 0.305 ± 0.456 | 82 | 0.196 ± 0.48 | 50 | 0.219 ± 0.679 | 38.9 | 0.347 ± 0.572 | 66 |
| *GGB4585 SGB6340* | 0.159 ± 0.288 | 46 | 0.067 ± 0.191 | 16 | 0 ± 0 | 5.6 | 0.112 ± 0.261 | 32 |
| *GGB9614 SGB15049* | 0.031 ± 0.046 | 64 | 0.013 ± 0.034 | 30 | 0.016 ± 0.041 | 38.9 | 0.016 ± 0.034 | 42 |
| *GGB9642 SGB15119* | 0.043 ± 0.051 | 50 | 0.045 ± 0.088 | 30 | 0.016 ± 0.049 | 11.1 | 0.044 ± 0.093 | 34 |
| *GGB9758 SGB15368* | 1.23 ± 1.377 | 74 | 0.282 ± 0.678 | 30 | 0.873 ± 1.594 | 55.6 | 0.903 ± 1.576 | 48 |
| *Oscillibacter sp ER4* | 0.803 ± 0.685 | 90 | 0.661 ± 0.943 | 62 | 0.717 ± 0.894 | 66.7 | 0.737 ± 0.93 | 72 |
| *Ruminococcus lactaris* | 0.204 ± 0.326 | 68 | 0.175 ± 0.309 | 42 | 0.068 ± 0.146 | 27.8 | 0.104 ± 0.159 | 52 |
| *Anaerotruncus colihominis* | 0.009 ± 0.032 | 18 | 0.017 ± 0.049 | 44 | 0.017 ± 0.037 | 50 | 0.014 ± 0.037 | 32 |
| *Anaerotruncus massiliensis* | 0.013 ± 0.061 | 12 | 0.022 ± 0.131 | 14 | 0.139 ± 0.397 | 50 | 0.009 ± 0.043 | 12 |
| *Blautia wexlerae* | 0.315 ± 0.386 | 98 | 0.765 ± 1.397 | 96 | 0.579 ± 0.551 | 100 | 0.423 ± 0.686 | 96 |
| *Butyricimonas faecalis* | 0.001 ± 0.006 | 4 | 0.006 ± 0.025 | 18 | 0.021 ± 0.067 | 22.2 | 0.004 ± 0.02 | 4 |
| *Christensenella hongkongensis* | 0 ± 0.001 | 2 | 0.001 ± 0.006 | 14 | 0.001 ± 0.002 | 33.3 | 0 ± 0.001 | 10 |
| *Clostridium scindens* | 0.001 ± 0.01 | 2 | 0.017 ± 0.058 | 22 | 0.005 ± 0.017 | 16.7 | 0.014 ± 0.053 | 12 |
| *Cuneatibacter caecimuris* | 0 ± 0.001 | 4 | 0.002 ± 0.009 | 22 | 0.015 ± 0.045 | 38.9 | 0.001 ± 0.004 | 12 |
| *Cuneatibacter sp NSJ 177* | 0 ± 0.001 | 10 | 0.001 ± 0.004 | 28 | 0.008 ± 0.021 | 50 | 0.001 ± 0.003 | 10 |
| *Dielma fastidiosa* | 0 ± 0.002 | 6 | 0.002 ± 0.008 | 16 | 0.003 ± 0.008 | 44.4 | 0.003 ± 0.011 | 12 |
| *Diplocloster agilis* | 0.001 ± 0.002 | 18 | 0.004 ± 0.013 | 36 | 0.008 ± 0.012 | 66.7 | 0.002 ± 0.005 | 32 |
| *Eisenbergiella tayi* | 0.008 ± 0.032 | 20 | 0.05 ± 0.146 | 36 | 0.032 ± 0.046 | 66.7 | 0.013 ± 0.05 | 24 |
| *Enterocloster bolteae* | 0.019 ± 0.067 | 28 | 0.109 ± 0.329 | 56 | 0.22 ± 0.394 | 77.8 | 0.159 ± 0.823 | 46 |
| *Enterocloster clostridioformis* | 0.034 ± 0.162 | 14 | 0.076 ± 0.218 | 40 | 0.036 ± 0.081 | 27.8 | 0.151 ± 0.831 | 22 |
| *Eubacteriaceae bacterium Marseille Q4139* | 0.001 ± 0.004 | 6 | 0.018 ± 0.073 | 26 | 0.019 ± 0.047 | 22.2 | 0.015 ± 0.061 | 14 |
| *Evtepia gabavorous* | 0.047 ± 0.089 | 34 | 0.069 ± 0.103 | 48 | 0.17 ± 0.181 | 77.8 | 0.086 ± 0.155 | 48 |
| *Flavonifractor plautii* | 0.161 ± 0.495 | 72 | 0.22 ± 0.477 | 90 | 0.256 ± 0.387 | 88.9 | 0.236 ± 0.382 | 80 |
| *GGB2982 SGB3964* | 0.012 ± 0.025 | 32 | 0.013 ± 0.023 | 40 | 0.033 ± 0.04 | 66.7 | 0.02 ± 0.032 | 48 |
| *GGB4583 SGB6334* | 0.001 ± 0.004 | 8 | 0.018 ± 0.05 | 26 | 0.001 ± 0.002 | 5.6 | 0.004 ± 0.017 | 12 |
| *GGB58233 SGB79883* | 0 ± 0.001 | 6 | 0.002 ± 0.004 | 26 | 0.003 ± 0.008 | 27.8 | 0.001 ± 0.003 | 20 |
| *GGB9522 SGB98319* | 0.001 ± 0.007 | 6 | 0.003 ± 0.009 | 14 | 0.009 ± 0.021 | 27.8 | 0.001 ± 0.005 | 8 |
| *GGB9719 SGB53514* | 0.002 ± 0.013 | 12 | 0.001 ± 0.003 | 8 | 0.076 ± 0.177 | 27.8 | 0.009 ± 0.037 | 8 |
| *Guopingia tenuis* | 0.01 ± 0.027 | 44 | 0.006 ± 0.01 | 52 | 0.021 ± 0.036 | 72.2 | 0.006 ± 0.008 | 60 |
| *Hungatella hathewayi* | 0.013 ± 0.045 | 18 | 0.18 ± 0.793 | 44 | 0.097 ± 0.347 | 61.1 | 0.022 ± 0.067 | 28 |
| *Intestinimonas butyriciproducens* | 0.054 ± 0.229 | 44 | 0.03 ± 0.051 | 56 | 0.054 ± 0.105 | 77.8 | 0.053 ± 0.15 | 60 |
| *Lachnotalea sp AF33 28* | 0.001 ± 0.003 | 30 | 0.005 ± 0.012 | 48 | 0.013 ± 0.029 | 55.6 | 0.004 ± 0.01 | 40 |
| *Lactococcus lactis* | 0.003 ± 0.018 | 8 | 0.002 ± 0.01 | 6 | 0.01 ± 0.021 | 33.3 | 0 ± 0.002 | 8 |
| *Massiliimalia timonensis* | 0 ± 0.001 | 4 | 0.001 ± 0.007 | 10 | 0.001 ± 0.003 | 27.8 | 0.001 ± 0.006 | 14 |
| *Ruminococcus gnavus* | 0.045 ± 0.12 | 60 | 0.125 ± 0.309 | 78 | 0.207 ± 0.464 | 83.3 | 0.729 ± 4.767 | 66 |
| *Ruthenibacterium lactatiformans* | 0.345 ± 0.78 | 94 | 0.259 ± 0.453 | 98 | 0.355 ± 0.438 | 100 | 0.305 ± 0.478 | 96 |

**Table S5. Combined functional pathway results from Maaslin2 and ANCOM-BC differential abundance analysis.** Group comparisons with nvHC as the reference. Covariates included gender and BMI. Pathways with q < 0.2 with either method in either evHC or iNPH groups are enlisted. All the pathways were enriched in evHC and/or iNPH, except for PWY-6700, denoted with ↓.

| Metacyc id | name | ancombc  q_evHC | Ancombc  q_iNPH | Maaslin2  q_evHC | Maaslin2  q_iNPH |
| --- | --- | --- | --- | --- | --- |
| **Carbohydrate Metabolism** | | | | | |
| ANAEROFRUCAT-PWY | homolactic fermentation | 1 | 1 | ≤ 0.2 | 0.4455 |
| GLYCOLYSIS | glycolysis I (from glucose 6-phosphate) | 1 | 1 | ≤ 0.2 | 0.46053 |
| NONOXIPENT-PWY | pentose phosphate pathway (non-oxidative branch) I | 1 | 1 | ≤ 0.2 | 0.77473 |
| PWY-5484 | glycolysis II (from fructose 6-phosphate) | 1 | 1 | ≤ 0.2 | 0.45796 |
| PWY-8178 | pentose phosphate pathway (non-oxidative branch) II | 1 | 1 | ≤ 0.2 | 0.87158 |
| P461-PWY | hexitol fermentation to lactate, formate, ethanol and acetate | 0.32154 | ≤ 0.01 | 0.20382 | ≤ 0.2 |
| **Amino Acid Metabolism** | | | | | |
| HOMOSER-METSYN-PWY | L-methionine biosynthesis I | 0.59143 | ≤ 0.01 | ≤ 0.2 | 0.23858 |
| MET-SAM-PWY | superpathway of S-adenosyl-L-methionine biosynthesis | 1 | ≤ 0.01 | ≤ 0.2 | 0.2364 |
| METSYN-PWY | superpathway of L-homoserine and L-methionine biosynthesis | 1 | ≤ 0.01 | ≤ 0.2 | 0.2364 |
| P4-PWY | superpathway of L-lysine, L-threonine and L-methionine biosynthesis I | 1 | ≤ 0.001 | 0.21008 | 0.2112 |
| PWY-5347 | superpathway of L-methionine biosynthesis (transsulfuration) | 1 | ≤ 0.01 | ≤ 0.2 | 0.2364 |
| PWY-6922 | L-Nδ-acetylornithine biosynthesis | 1 | ≤ 0.05 | 0.50499 | ≤ 0.2 |
| SER-GLYSYN-PWY | superpathway of L-serine and glycine biosynthesis I | 1 | 1 | ≤ 0.2 | 0.28463 |
| PWY0-781 | aspartate superpathway | 1 | ≤ 0.001 | 0.21008 | 0.2112 |
| **Lipid Metabolism** | | | | | |
| PWY-5136 | fatty acid β-oxidation II (plant peroxisome) | 0.49838 | ≤ 0.05 | ≤ 0.2 | ≤ 0.2 |
| PWY-5367 | petroselinate biosynthesis | 1 | 1 | ≤ 0.2 | 0.54271 |
| PWY-6284 | superpathway of unsaturated fatty acids biosynthesis (E. coli) | 1 | 1 | ≤ 0.2 | 0.48779 |
| PWY-6285 | superpathway of fatty acids biosynthesis (E. coli) | 1 | 1 | ≤ 0.2 | 0.54271 |
| PWY-7409 | phospholipid remodeling (phosphatidylethanolamine, yeast) | 1 | 1 | ≤ 0.2 | 0.45796 |
| **Nucleotide Metabolism** | | | | | |
| P164-PWY | purine nucleobases degradation I (anaerobic) | 1 | 1 | ≤ 0.2 | ≤ 0.2 |
| PWY-6353 | purine nucleotides degradation II (aerobic) | 1 | 1 | ≤ 0.2 | 0.37714 |
| PWY-6606 | guanosine nucleotides degradation II | 0.95204 | 1 | ≤ 0.1 | 0.27417 |
| PWY-6608 | guanosine nucleotides degradation III | 1 | 1 | ≤ 0.2 | 0.20021 |
| PWY0-1298 | superpathway of pyrimidine deoxyribonucleosides degradation | 1 | 1 | ≤ 0.2 | 0.35821 |
| **Energy Metabolism** | | | | | |
| PWY-5676 | acetyl-CoA fermentation to butanoate II | 1 | 0.35687 | ≤ 0.2 | ≤ 0.2 |
| PWY-5677 | succinate fermentation to butanoate | 1 | 0.2185 | 0.34459 | ≤ 0.2 |
| PWY-7384 | anaerobic energy metabolism (invertebrates, mitochondrial) | 1 | 1 | ≤ 0.2 | 0.67148 |
| PWY-7389 | superpathway of anaerobic energy metabolism (invertebrates) | 1 | 1 | ≤ 0.2 | 0.6754 |
| PWY-7385 | 1,3-propanediol biosynthesis (engineered) | ≤ 0.05 | 1 | ≤ 0.2 | 0.77288 |
| **Cofactor and Vitamin Metabolism** | | | | | |
| HEME-BIOSYNTHESIS-II | heme b biosynthesis I (aerobic) | 1 | 1 | ≤ 0.2 | 0.41554 |
| HEMESYN2-PWY | heme b biosynthesis II (oxygen-independent) | ≤ 0.2 | ≤ 0.05 | ≤ 0.2 | 0.30316 |
| PWY-5918 | superpathway of heme b biosynthesis from glutamate | 1 | ≤ 0.2 | 0.28935 | 0.27222 |
| PWY-6612 | superpathway of tetrahydrofolate biosynthesis | 1 | 1 | ≤ 0.2 | 0.30889 |
| **↓** PWY-6700 | queuosine biosynthesis I (de novo) | 1 | 1 | ≤ 0.2 | 0.40138 |
| PWY-6708 | ubiquinol-8 biosynthesis (early decarboxylation) | 1 | 1 | ≤ 0.2 | 0.29818 |
| PWY-6892 | thiazole component of thiamine diphosphate biosynthesis I | 1 | 1 | ≤ 0.2 | 0.30889 |
| PWY-7204 | pyridoxal 5-phosphate salvage II (plants) | 1 | ≤ 0.05 | 0.20021 | ≤ 0.2 |
| UBISYN-PWY | superpathway of ubiquinol-8 biosynthesis (early decarboxylation) | 1 | 1 | ≤ 0.2 | 0.29818 |
| **Xenobiotics Biodegradation and Metabolism** | | | | | |
| P161-PWY | acetylene degradation (anaerobic) | 0.20019 | ≤ 0.0001 | ≤ 0.2 | ≤ 0.2 |
| PWY-7805 | (aminomethyl)phosphonate degradation | 0.66787 | 1 | ≤ 0.2 | 0.37424 |
| PWY-7807 | glyphosate degradation III | 0.50902 | 1 | ≤ 0.2 | 0.35806 |
| PWY-7873 | D-erythronate degradation II | 1 | 1 | ≤ 0.2 | 0.54793 |
| PWY-7874 | L-threonate degradation | ≤ 0.1 | 1 | ≤ 0.2 | 0.52378 |
| PWY-8131 | 5-deoxyadenosine degradation II | 1 | 1 | 0.34459 | ≤ 0.2 |
| PWY-6992 | 1,5-anhydrofructose degradation | 1 | 0.28418 | 0.29838 | ≤ 0.2 |
| PWY-7013 | (S)-propane-1,2-diol degradation | 1 | 1 | ≤ 0.2 | 0.31851 |
| PWY0-1533 | methylphosphonate degradation I | 0.43888 | 1 | ≤ 0.2 | 0.35821 |
| **Allantoin Metabolism** | | | | | |
| PWY-5692 | allantoin degradation to glyoxylate II | ≤ 0.01 | 0.83552 | ≤ 0.05 | 0.21139 |
| PWY0-41 | allantoin degradation IV (anaerobic) | ≤ 0.2 | 1 | ≤ 0.2 | 0.45796 |
| URDEGR-PWY | superpathway of allantoin degradation in plants | ≤ 0.01 | 0.83552 | ≤ 0.05 | 0.21139 |
| **Galactarate and Glucarate Metabolism** | | | | | |
| GALACTARDEG-PWY | D-galactarate degradation I | 0.23086 | 1 | ≤ 0.2 | 0.30889 |
| GLUCARDEG-PWY | D-glucarate degradation I | ≤ 0.2 | 1 | ≤ 0.2 | 0.27369 |
| GLUCARGALACTSUPER-PWY | superpathway of D-glucarate and D-galactarate degradation | 0.23086 | 1 | ≤ 0.2 | 0.30889 |
| **Other Metabolic Pathways** | | | | | |
| METHGLYUT-PWY | superpathway of methylglyoxal degradation | ≤ 0.05 | ≤ 0.001 | ≤ 0.2 | ≤ 0.2 |
| P441-PWY | superpathway of N-acetylneuraminate degradation | 1 | ≤ 0.0001 | 0.37424 | ≤ 0.2 |
| PWY0-1586 | peptidoglycan maturation (meso-diaminopimelate containing) | 1 | 1 | ≤ 0.2 | 0.63357 |
| PWY4LZ-257 | superpathway of fermentation (Chlamydomonas reinhardtii) | 0.24944 | ≤ 0.0001 | ≤ 0.2 | ≤ 0.2 |
| POLYAMSYN-PWY | superpathway of polyamine biosynthesis I | 1 | 1 | ≤ 0.2 | 0.54418 |
| PWY-6478 | GDP-D-glycero-α-D-manno-heptose biosynthesis | 1 | 1 | 0.27369 | ≤ 0.2 |
| PWY-7159 | 3,8-divinyl-chlorophyllide a biosynthesis III (aerobic, light independent) | 1 | 1 | 0.45796 | ≤ 0.2 |

**Table S6. Current knowledge on species identified in this study.** Short description of the studies enlisted: Sun et al analyzed datasets containing samples of 3728 patients with 28 different disease or unhealthy statuses; Gacesa et al associated microbiome features with self-reported health and 81 diseases of 8208 individuals; Su et al modeled data from 2,320 individuals with either healthy or 8 different disease states; Gupta et al analyzed 4347 human stool metagenomes across healthy and 12 different nonhealthy conditions and defined 7 species as health-prevalent and 43 species as health-scarce; Lee et al conducted association studies of 43 cohorts from 23 different diseases, totaling 2185 samples. ↓ and ↑ signs in column “Direction” denote respectively species decreased or increased in iNPH and/or evHC groups compared to nvHC. + and – signs denote respectively whether the species were identified health or disease- associated in the referenced studies. Differentially abundant species enlisted in Table S3 that were not identified in the listed studies were omitted from the table.

| **Species** | **Direction** | (Sun et al. 2024) | (Gacesa et al. 2022) | (Su et al. 2022) | (Gupta et al. 2020) | (Lee et al. 2024) |
| --- | --- | --- | --- | --- | --- | --- |
| *Clostridiaceae bacterium Marseille Q4145, SGB4769* | ↓ | + |  |  |  |  |
| *Clostridium sp AF20 17LB, SGB4714* | ↓ | + |  |  |  |  |
| *Collinsella aerofaciens* | ↓ |  |  | + |  |  |
| *Fusicatenibacter saccharivorans* | ↓ | + |  | + |  |  |
| *GGB4585 SGB6340* | ↓ | + |  |  |  |  |
| *GGB9614 SGB15049* | ↓ | + |  |  |  |  |
| *GGB9758 SGB15368* | ↓ | + |  |  |  |  |
| *Oscillibacter sp ER4, SGB15254* | ↓ | + |  |  |  |  |
| *Ruminococcus lactaris* | ↓ | + | + | + |  |  |
| *Anaerotruncus colihominis* | ↑ |  | - |  | - |  |
| *Butyricimonas faecalis* | ↑ | + |  |  |  |  |
| *Clostridium scindens* | ↑ | - |  |  |  |  |
| *Dielma fastidiosa* | ↑ |  |  | - |  |  |
| *Eisenbergiella tayi* | ↑ | - |  |  |  |  |
| *Enterocloster bolteae* | ↑ | - | - | - | - | - |
| *Enterocloster clostridioformis* | ↑ | - |  |  | - | - |
| *Flavonifractor plautii* | ↑ | - | - | - | - | - |
| *GGB2982 SGB3964* | ↑ | + |  |  |  |  |
| *Hungatella hathewayi* | ↑ | - |  | - | - |  |
| *Lactococcus lactis* | ↑ |  |  | + |  |  |
| *Massiliimalia timonensis* | ↑ | - |  |  |  |  |
| *Ruminococcus gnavus* | ↑ | - | - | - | - |  |
| *Ruthenibacterium lactatiformans* | ↑ |  |  | + |  |  |
