# Supplementary material for: Gut microbiome signatures in iNPH: Insights from a shotgun metagenomics study
Source: PLoS One. 2025 Sep 15;20(9):e0330251. doi: 10.1371/journal.pone.0330251 (PMC12435679; doi:10.1371/journal.pone.0330251)
Supplement: Attachment — Submitted filename: comments.docx [file pone.0330251.s004.docx]

Reviewer Comments

This study provides novel insights into gut microbiome signatures in iNPH using shotgun metagenomics. The findings are promising but would benefit from the instructions below:

(1)The sample size of the iNPH group (n=18) is relatively small, which may limit the statistical power and generalizability of the findings. The authors should explicitly acknowledge this limitation and suggest that future studies with larger cohorts are needed for validation.

(2)Were potential confounding factors (e.g., diet, medication use, comorbidities) adequately controlled for? Since these factors can influence gut microbiota composition, their absence in the analysis may affect the interpretation of results.

(3)The rationale for selecting Maaslin2 and ANCOM-BC for differential abundance analysis should be clarified. Given that microbiome data are compositional, how were false discovery rates (FDR) and multiple testing corrections handled?

(4)The functional analysis suggests enrichment of pathways like S-adenosylmethionine (SAM) biosynthesis, but are these findings supported by independent validation (e.g., metabolomics)? If not, this should be discussed as a limitation.

(5)Several microbial taxa (e.g., *Ruminococcus gnavus*, *Enterocloster bolteae*) have been linked to other neurological disorders (e.g., Alzheimer’s, Parkinson’s). How specific are these signatures to iNPH? A comparative discussion with existing literature would strengthen the novelty of the findings.

(6)The correlation between microbial taxa and clinical traits (e.g., ventricular volume, gait disturbance) is intriguing, but can the authors speculate on potential causal mechanisms? For example, could gut dysbiosis contribute to neuroinflammation and CSF dynamics, or vice versa?

(7)The study identifies potential microbial biomarkers for iNPH, but their diagnostic or therapeutic utility remains unclear. Could these findings be translated into clinical applications (e.g., microbiome-based diagnostics or probiotics)?

(8)The gut-brain axis hypothesis is central to this work, but the discussion could be expanded to include possible pathways (e.g., SCFA production, immune modulation, barrier dysfunction).

(9)Figure 3 (barplot of microbial taxa): Consider adding a legend to clarify the direction of change (enriched/depleted) and significance thresholds.

(10)Figure 4 (correlation heatmap): Some labels are unclear—could the axes be better annotated? Additionally, were correlation p-values adjusted for multiple comparisons?

(11)Some sentences are overly complex (e.g., in the Abstract and Discussion). Simplifying phrasing would improve readability.

(12)Ensure consistent formatting of microbial names (e.g., *Eviepia gabavorous* should be italicized throughout).
