# Supplementary material for: Gut microbiome signatures in iNPH: Insights from a shotgun metagenomics study
Source: PLoS One. 2025 Sep 15;20(9):e0330251. doi: 10.1371/journal.pone.0330251 (PMC12435679; doi:10.1371/journal.pone.0330251)
Supplement: Attachment — Submitted filename: Response to Reviewers.docx [file pone.0330251.s006.docx]

**Response to Reviewers**

We thank the reviewers for their thoughtful and detailed feedback. We have addressed each point below and have made corresponding revisions or clarifications to the manuscript as indicated.

Journal Requirements:

1. When submitting your revision, we need you to address these additional requirements.

Please ensure that your manuscript meets PLOS ONE's style requirements, including those for file naming. The PLOS ONE style templates can be found at

https://journals.plos.org/plosone/s/file?id=wjVg/PLOSOne_formatting_sample_main_body.pdf and

<https://journals.plos.org/plosone/s/file?id=ba62/PLOSOne_formatting_sample_title_authors_affiliations.pdf>

**Response:**
We have reviewed the manuscript to ensure it adheres to the PLOS ONE style requirements, including file naming conventions, as per the provided templates.

2. Please describe in your methods section how capacity to provide consent was determined for the participants in this study. Please also state whether your ethics committee or IRB approved this consent procedure. If you did not assess capacity to consent please briefly outline why this was not necessary in this case.

**Response:**

Added to manuscript lines 124-129: “When a legal representative was available, the study design, potential risks, and benefits were discussed with them. In the absence of a legal representative, the participant’s capacity to understand the study design, risks, and benefits was assessed clinically. Participants with mild or severe dementia were always accompanied by a caregiver, and study participation was discussed jointly with both the participant and the caregiver. “

This approach was deemed appropriate given the minimal-risk nature of the study and was consistent with clinical standards for capacity assessment, although it was not formally approved as a separate procedure by the ethics committee.

3. Thank you for stating the following financial disclosure: [This study has been funded by Race Against Dementia, UK (Charity Number: 1165559)].

Please state what role the funders took in the study. If the funders had no role, please state: "The funders had no role in study design, data collection and analysis, decision to publish, or preparation of the manuscript."

If this statement is not correct you must amend it as needed.

Please include this amended Role of Funder statement in your cover letter; we will change the online submission form on your behalf.

**Response:** "The funders had no role in study design, data collection and analysis, decision to publish, or preparation of the manuscript." – the statement is correct.

4. When completing the data availability statement of the submission form, you indicated that you will make your data available on acceptance. We strongly recommend all authors decide on a data sharing plan before acceptance, as the process can be lengthy and hold up publication timelines. Please note that, though access restrictions are acceptable now, your entire data will need to be made freely accessible if your manuscript is accepted for publication. This policy applies to all data except where public deposition would breach compliance with the protocol approved by your research ethics board. If you are unable to adhere to our open data policy, please kindly revise your statement to explain your reasoning and we will seek the editor's input on an exemption. Please be assured that, once you have provided your new statement, the assessment of your exemption will not hold up the peer review process.

**Response:** All raw sequencing data generated and analyzed during this study will be made publicly available at EBI ENA. The request to release them has been made today.

Reviewer #1: Reviewer Comments
This study provides novel insights into gut microbiome signatures in iNPH using shotgun metagenomics. The findings are promising but would benefit from the instructions below:
(1)The sample size of the iNPH group (n=18) is relatively small, which may limit the statistical power and generalizability of the findings. The authors should explicitly acknowledge this limitation and suggest that future studies with larger cohorts are needed for validation.

**Response:**
We fully agree that the relatively small sample size is a limitation. Recruiting iNPH patients is inherently challenging due to the difficulty in diagnosis and the fact that many patients are fatigued or overwhelmed, reducing their willingness to participate in research. Despite these challenges, we believe the results offer valuable early insights and warrant further investigation.

**Added to manuscript:**

Lines 505-507

“We acknowledge that patient recruitment for iNPH is particularly challenging due to the diagnostic complexity of the disease and the physical and psychological burden on patients, which affects research participation.”

(2)Were potential confounding factors (e.g., diet, medication use, comorbidities) adequately controlled for? Since these factors can influence gut microbiota composition, their absence in the analysis may affect the interpretation of results.

**Response:**
While dietary and medication data were collected, especially for iNPH patients and subjects with enlarged ventricles, the corresponding data for AD and healthy controls are not uniformly stored and remain largely in non-analyzable formats. As such, these variables were not included in the current analysis, which we acknowledge as a limitation.

**Added to manuscript:**

Lines 510-512

" Although these data were partially collected, they were not available in complete form or suitable format for reliable analysis across all study groups at this stage. Future studies will prioritize harmonized collection and analysis of these important variables."

(3)The rationale for selecting Maaslin2 and ANCOM-BC for differential abundance analysis should be clarified. Given that microbiome data are compositional, how were false discovery rates (FDR) and multiple testing corrections handled?

**Response:**
We selected Maaslin2 and ANCOM-BC as both are widely used and reliable methods for differential abundance testing in microbiome studies, and they are designed to address the compositional nature of microbiome data. Multiple testing correction was applied using the default settings in each tool: Benjamini-Hochberg (BH) method for Maaslin2 and Holm method for ANCOM-BC. We have now clarified this in the Methods section.

**Added to manuscript:**

Lines 228-231

" These are widely adopted and validated methods tailored for compositional microbiome data (38). The Maaslin2 and ANCOM-BC results were corrected for multiple testing using their respective default methods, the Benjamini-Hochberg (BH) procedure and the Holm method."

(4)The functional analysis suggests enrichment of pathways like S-adenosylmethionine (SAM) biosynthesis, but are these findings supported by independent validation (e.g., metabolomics)? If not, this should be discussed as a limitation.

**Response:**
We agree. The current study uses predictive functional profiling based on metagenomic data, and we do not have corresponding metabolomics to confirm pathway activity. This has been added as a limitation in the discussion.

**Added to manuscript:**

Lines 499-502

" Functional predictions were derived from metagenomic profiles without direct metabolomic validation, which constitutes a limitation of the present study. Future work incorporating untargeted metabolomics will be essential to confirm the biological relevance of these pathway enrichments."

(5)Several microbial taxa (e.g., Ruminococcus gnavus, Enterocloster bolteae) have been linked to other neurological disorders (e.g., Alzheimer’s, Parkinson’s). How specific are these signatures to iNPH? A comparative discussion with existing literature would strengthen the novelty of the findings.

**Response:**
Thank you for raising this point. We have addressed it by expanding the discussion, where we compare enriched taxa in iNPH with those reported in other disorders. While some overlaps exist (e.g., R. gnavus), we also highlight species that appear more specific to iNPH based on their correlation with disease traits (lines 446-450).

**Added to manuscript:**

Lines 443-445

" Some enriched taxa, such as R. gnavus, which has been reported in studies of cognitive decline and Parkinson’s disease, have been associated with neurocognitive disorders, although findings remain inconsistent and often context-dependent (49,50)”

(6)The correlation between microbial taxa and clinical traits (e.g., ventricular volume, gait disturbance) is intriguing, but can the authors speculate on potential causal mechanisms? For example, could gut dysbiosis contribute to neuroinflammation and CSF dynamics, or vice versa?

**Response:**
We agree and have expanded the discussion.

**Added to manuscript:**

Lines 468-481

" Recent findings have shown that germ-free mice exhibit increased permeability of both the blood–brain barrier (BBB) and the blood–cerebrospinal fluid (CSF) barrier, accompanied by disrupted expression and subcellular localization of tight junction proteins (64,65). These structural alterations were reversible following recolonization with a normal gut microbiota or supplementation with short-chain fatty acids (SCFAs), highlighting the microbiota’s critical role in maintaining barrier function in the central nervous system. Such barrier dysregulation may contribute to impaired CSF homeostasis and immune cell infiltration, mechanisms potentially relevant to the ventricular enlargement and inflammation observed in iNPH (66). Furthermore, gut dysbiosis has been implicated in chronic neuroinflammation, protein misfolding, and impaired clearance pathways—features common to neurodegenerative conditions including Alzheimer’s and Parkinson’s disease (67). However, to the best of our knowledge, the functional dysregulation occurring in iNPH remains completely elusive. Here, we show alterations in microbial signatures in iNPH that may reflect disturbances in gut–brain communication, influencing barrier permeability and neuroimmune signaling."

(7)The study identifies potential microbial biomarkers for iNPH, but their diagnostic or therapeutic utility remains unclear. Could these findings be translated into clinical applications (e.g., microbiome-based diagnostics or probiotics)?

**Response:**
We agree this is a promising area for translational research. However, we remain cautious about drawing premature conclusions. Larger, longitudinal studies will be essential to assess the clinical utility of identified microbial signatures for diagnostics or therapeutic modulation.

**Added to manuscript:**

Lines 518-521

" While the identification of disease-associated and health-linked microbial taxa is encouraging, the diagnostic or therapeutic utility of these findings remains to be validated. Future work incorporating longitudinal microbiome tracking, dietary and medication data, and targeted interventions will be key to exploring microbiome-based diagnostics or therapeutics. "

(8)The gut-brain axis hypothesis is central to this work, but the discussion could be expanded to include possible pathways (e.g., SCFA production, immune modulation, barrier dysfunction).

**Response:**
We have expanded the discussion to include key gut–brain communication pathways. A detailed mechanistic analysis remains beyond the scope of this article, but our findings align with emerging evidence on SCFA-mediated signaling, immune modulation, and barrier function.

**Added to manuscript:**

Lines 466-468

" Gut-brain communication may be mediated via multiple pathways, including SCFA production, immune modulation, and maintenance of epithelial and endothelial barrier function along the gut–brain axis (63)."

(9)Figure 3 (barplot of microbial taxa): Consider adding a legend to clarify the direction of change (enriched/depleted) and significance thresholds.

**Response:**
We appreciate this suggestion and will revise Figure 3 to include a legend clarifying the directionality of changes and the thresholds for statistical significance.

**Added to manuscript:**

Line 353-354 : (positive values indicate enrichment, negative values indicate depletion). The figure was amended to include the stars denoting significance.

(10)Figure 4 (correlation heatmap): Some labels are unclear—could the axes be better annotated? Additionally, were correlation p-values adjusted for multiple comparisons?

**Response:**

We appreciate the reviewer’s comment. The apparent lack of clarity in some axis labels may be due to image compression in the submission PDF, which can cause blurring. In the original high-resolution TIFF file prepared for publication, all labels are clearly legible. Regarding p-values, we confirm that correlation p-values were adjusted for multiple comparisons using the Benjamini-Hochberg (BH) procedure, as noted in both the figure legend (“adjusted p-value”) and the Methods section under *Statistical analysis*.

(11)Some sentences are overly complex (e.g., in the Abstract and Discussion). Simplifying phrasing would improve readability.

We appreciate the reviewer’s comment. We have simplified select sentences in the Abstract and Discussion to improve clarity, including lines 33-34 and 36-38 in the Abstract and lines 406-407 and 421 in the Discussion. These minor edits aim to enhance readability without altering the scientific meaning.

(12)Ensure consistent formatting of microbial names (e.g., Eviepia gabavorous should be italicized throughout).

**Response:**
Thank you for noting this. We have carefully reviewed the manuscript to ensure consistent italicization of all microbial species names.

Reviewer #2: In this research, shotgun metagenomics was employed to conduct an in - depth analysis of the gut microbiome in 18 patients with iNPH. The results were then contrasted with those of 50 healthy controls, 50 individuals presenting with ventriculomegaly yet without iNPH symptoms, and 50 Alzheimer's disease patients.

The outcomes of this study indicate that there are unique gut microbiome signatures associated with iNPH. These signatures provide valuable insights into the potential interactions between the gut and the brain, which may play a role in the pathophysiology of iNPH. Moreover, they also pinpoint possible targets that could be exploited in future therapeutic strategies for this disorder.

However, several aspects still need to be further improved.

1.The sample size of iNPH patients is relatively small, which may limit the generalizability and reliability of the research findings. A larger sample size would be beneficial to more accurately reflect the real situation of the iNPH population.

**Response:**
Please refer to our response to Reviewer 1, point (1). Recruitment of iNPH patients remains challenging, but we acknowledge the limitation.

2.Additionally, the lack of detailed information on dietary intake and medication use restricts a comprehensive understanding of the influencing factors of gut microbiome changes. It is essential to collect such information to better interpret the relationship between external factors and gut microbiome alterations.

**Response:**
Please refer to our response to Reviewer 1, point (2). While some data were collected, they were not uniformly available and thus omitted in this initial analysis.

3.Finally, the cross - sectional nature of this study makes it difficult to determine the causal relationship between gut microbiome changes and the onset or progression of iNPH. Longitudinal studies are needed to track the development of individuals over time and clarify these causal links.

**Response:**
We agree entirely. To address this limitation, a longitudinal study is currently ongoing in our research group. The importance of longitudinal data has also been emphasized in the revised manuscript (line 415, 516).

**Added to manuscript:**

Line 416-417

" A longitudinal follow-up study is ongoing and may help to clarify these trajectories."
